# Commentary: Transcriptome analysis of host inflammatory responses to the ectoparasitic mite *Sarcoptes scabiei* var. *hominis*

**DOI:** 10.3389/fimmu.2023.1128688

**Published:** 2023-02-15

**Authors:** Kate E. Mounsey

**Affiliations:** ^1^ School of Health, University of the Sunshine Coast, Maroochydore, QLD, Australia; ^2^ Sunshine Coast Health Institute, Birtinya, QLD, Australia

**Keywords:** scabies, *Sarcoptes scabiei*, inflammatory responses, host-parasite interactions, immunology

## 1 Introduction

Scabies is a highly prevalent infectious disease of underappreciated health burden. In resource poor settings, secondary infections of scabietic lesions are linked to significant sequelae including acute post-streptococcal glomerulonephritis and rheumatic heart disease (1). Crusted scabies, a rare clinical manifestation associated with hyperproliferation of mites, is associated with high mortality if untreated, and sarcoptic mange in animals is a significant health and welfare issue (2). While scabies is readily treatable in individuals, timely diagnosis is confounded due to a lack of sensitive and specific methods. Emerging drug resistance is a concern in some regions (3), and population-level control can be difficult to implement (4).

There are few studies on the immunology of scabies, and those that do exist are mostly based on small case numbers, lack appropriate controls, or focus on a small number of candidate molecules (5). Relative to other parasitic diseases, scabies is especially difficult to study due to the inability to maintain mites in culture, and a historical lack of tractable animal models for *in-vivo* study (6). Access to infested patients for research can be difficult, especially for more invasive procedures such as skin biopsy collection. This is important, as scabies mites are limited to the host epidermis, and studying localised immune responses in the skin provides rich insight compared to peripheral blood.

In this paper, Shehwana, Ijaz and colleagues describe the first transcriptomic study on scabietic lesions from human patients (n = 5) and healthy controls (n=3). They also conduct a detailed comparative analysis of their results with that of previous studies- including an *in-vivo* transcriptomic analysis of skin biopsies from infested pigs published by my group (7), and *in-vitro* mite infested human skin equivalents (8). From this, a clearer and more consistent picture of the immune and inflammatory events associated with scabies begin to emerge. In this commentary, I discuss key themes emerging from this paper, and its contribution to this neglected aspect of parasite immunology.

## 2 Timing of scabies immune responses

Scabies involves a delayed onset of clinical manifestations, due to modulation of inflammatory responses by mite molecules. The delay is estimated to be four to six weeks in a primary exposure and reduces with repeat infestations- consistent with the acquisition of immunological memory. In the study by Shehwana, Ijaz et al., clinical history of the patients is not provided. Patients are described as “one to two weeks post-exposure”- it is uncertain whether this actually refers to a defined exposure event, or rather the onset of clinical manifestations. If the latter, it is unsurprising that the human transcriptome results aligned best with the porcine samples from week 8 post-infestation. If the former, they could potentially represent a repeat exposure given the rapidity of symptom onset.

In our porcine study we found variation in gene expression profiles between pre-clinical (1-2 weeks), and later (week 8) infestation, with a switch from suppressed to activated inflammatory responses. In the study by Shehwana, Ijaz et al., the authors also observed distinct clustering according to time. This temporality in response is important to consider in the design and interpretation of other studies and especially where diagnostic biomarkers for early diagnosis are evaluated. It may also partially explain areas of discordance between the different datasets. This is most apparent in the gene expression signatures relating to T cell activation and proliferation. In the human samples, downregulation of genes related to immunoglobulin and TCR binding was observed, whereas in the porcine data these genes were downregulated in earlier weeks but upregulated at week 8. This could also be related to clinical severity, as skin T cell proliferation, especially CD8+ and γδ−T cells has been observed in crusted relative to ordinary scabies (9, 10).

## 3 Key players of scabies-associated skin inflammation

Scabies shares clinical features with aspects of atopic dermatitis and psoriasis- both are common differential diagnoses for scabies. Being an ectoparasite, it also shares immunomodulatory features with other mucosal parasites- *in-vitro* studies show the inhibition of complement (11) and stimulation of regulatory T cells (12) by mite molecules. However, *in-vivo*, it was markers of skin inflammation that are most apparent. Clear patterns between the human and porcine datasets were observed, including activation of JAK/STAT inflammatory signaling via IL-19 and IL-20, and T cell chemokine signatures. CXCL8, chemoattractant for neutrophils and a strong stimulator of inflammation was one of the most highly upregulated genes in both datasets. Also highly expressed were the S100 family of antimicrobial peptides, as seen in other proliferative skin disorders. Other upregulated genes linked to a psoriatic gene expression profile included DEFB4A, IL17A, and IL17RB. It is of note that several of these key genes were not seen in the human skin equivalent dataset, reinforcing the importance of *in-vivo* studies to obtain a comprehensive picture of immune responses for descriptive studies.

## 4 Molecules associated with clinical severity of scabies

It was of interest to compare and contrast the magnitude of gene expression between ordinary (human) and crusted (porcine) datasets. Crusted scabies was associated with much higher expression of CXCL8, IL-19, IL-20, IL-17A, S100A8 and S100A12. Immunotherapy targeting IL-17A, which has downstream impacts on IL-19 and IL-20 and subsequent keratinocyte proliferation (13, 14) is now widely utilized in psoriasis and may be useful in ameliorating crusted scabies. Highly upregulated genes in the porcine crusted dataset but not the human dataset included arginase 1 and CXCL6. The authors noted AADACL3 downregulation and its previous association with crusted scabies *via* chromosomal duplication and altered expression (15). This gene was not represented in the porcine dataset and any association should be interpreted with caution given it is only based on one case study with functional links unclear.

## 5 Reframing old paradigms of scabies immunology

Previous studies on scabies based on measurement of cytokines in peripheral blood stimulated with mite antigens have characterized ordinary scabies as a Th1 response, and crusted scabies as a non-protective, Th2 response (16). A recent meta-analysis (5) describes sarcoptic mange and evidence supporting either Type I or Type IV hypersensitivity, and unfortunately excluded studies on humans and the transcriptomic studies described herein. Such compartmentalization is too generalist. The studies by Shewana, Ijaz et al., and our group has revealed that both ordinary and crusted scabies are associated with Th2 signatures (IL-4 and IL-13), as would be expected from a parasitic infestation, with an increased magnitude of IL-17 pathway related cytokines and effector molecules in crusted scabies (10, 17). As crusted scabies is very commonly associated with extremely high IgE and often eosinophilia (18), but also shows T cell dysregulation in the skin, elements of both Type I and Type IV hypersensitivity are represented.

## 6 Conclusion

The authors are to be commended on this important study and its contribution to our limited understanding of scabies immunopathology. While much work needs to be done to identify specific deficits and factors impacting clinical severity of scabies, we now have a clearer picture of biological pathways, candidate biomarkers and immunotherapeutic targets for this neglected disease. Future studies focused on crusted scabies in humans, mechanisms of immunomodulation via host-parasite interactions, and time-associated immune profiling would add further contributions. The strong concordance between the human and porcine data attests to the utility of the porcine model to inform elements of human scabies.

## Author contributions

The author confirms being the sole contributor of this work and has approved it for publication.
